# Progress

**Published:** 1871-09

**Authors:** 


### PROGRESS.

Dr. W. F. Westmoreland, now on a visit to the Northern and Western cities, writes from New York in a private letter that he is securing a corps of contributors to the "JOURNAL," both North and West, composed of the most prominent medical men in the country, whose contributions, with others which will be obtained from many of the leading physicians of the South, must place it at once in the front rank of medical periodicals.

He also states that he has perfected arrangements in New York with an engraver, by which the "JOURNAL" will be thoroughly illustrated, and contributors may rely upon every advantage that they can possibly desire in this way, free from any charge whatever.

He feels highly gratified by the cordial reception he has met, and is assured that both the "JOURNAL" and the Atlanta Academy of Medicine have attracted favorable attention, and are welcomed as valuable co-laborers in advancing medical science. While, however, it has been regarded as essential to the success determined upon for the ATLANTA MEDICAL AND SURGICAL JOURNAL, that a regular corps of the leading medical men of the country should be obtained to contribute to its columns, it has not been, by any means, the intention or desire to exclude any respectable medical gentleman who is willing to favor us with a paper, and contributions from all such are earnestly solicited, and will receive the same courtesy as those from the highest in the land.

Upon the return of Dr. W. F. Westmoreland, and in the next number of the "JOURNAL," an announcement will be made of arrangements for putting the "JOURNAL" upon the high basis to which reference has been made.

